# Norovirus in children under 2 years of age: an epidemiological study in Panama during the COVID-19 pandemic

**DOI:** 10.3389/fped.2024.1292967

**Published:** 2024-02-15

**Authors:** Rodrigo DeAntonio, Morgan Hess-Holtz, Leyda Abrego, Zeuz Capitan-Barrios, Leyla Hernandez Donoso, Tirza De León, Xavier Sáez Llorens, Brechla Moreno, John Gerard Weil

**Affiliations:** ^1^ Centro de Vacunación e Investigación CEVAXIN, The Panama Clinic, Panama City, Panama; ^2^ Instituto Conmemorativo Gorgas de Estudios de la Salud, Departamento de Investigacion en Virologia y Biotecnologia, Panama City, Panama; ^3^ Departamento de Microbiología y Parasitología, Facultad de Ciencias Naturales, Exactas y Tecnología, Universidad de Panama, Panama City, Panama; ^4^ Takeda Pharmaceuticals International AG., Zurich, Switzerland; ^5^ Hospital Materno Infantil José Domingo de Obaldia, David, Panama; ^6^ Infectious Disease Department, Hospital del Niño Dr José Renán Esquivel, Panama City, Panama

**Keywords:** infectious diarrheal disease, norovirus, gastroenteritis, epidemiology, COVID-19

## Abstract

**Introduction:**

Norovirus infection is a common cause of acute gastroenteritis (AGE). Surveillance activities are important to aid investigation into effective norovirus control strategies, including vaccination. Here, we report ancillary findings related to the incidence, prevalence, and etiology of AGE caused by norovirus in Panama after adjustment of study methodology to comply with national coronavirus disease 2019 (COVID-19) mandates.

**Methods:**

In January 2020, children aged <2 years began enrolling into an epidemiological study in Panama to estimate the burden of norovirus in preparation for evaluating upcoming prevention strategies. This included an observational, longitudinal, community-based AGE surveillance study and a hospital-based AGE surveillance study. For the longitudinal study, healthy children aged 5–18 months were enrolled from January 6 through March 23, 2020, with a follow-up of approximately 6 months. The last participant was contacted on September 23, 2020. For the hospital-based study, starting on January 21, 2020, children aged <2 years who were admitted to the Hospital del Niño Dr. José Renán Esquivel in Panama City due to AGE were evaluated. The last sample was collected on September 29, 2020. Collected stool samples were tested for norovirus as well as astrovirus, sapovirus, and various enteropathogens. Unfortunately, this study was disrupted by the subsequent implementation of disease transmission control procedures for the COVID-19 pandemic, and the study methodology was revised to comply with COVID-19 mandates.

**Results:**

In the longitudinal surveillance cohort [*N* = 400 (Chiriquí, *n* = 239; Panama, *n* = 161)], a total of 185 AGE episodes were documented (Chiriquí, *n* = 85; Panama, *n* = 100) resulting in an overall AGE incidence of 11.6 (95% CI: 9.99–13.4) episodes per 100 child-months. The norovirus-related AGE incidence was 0.3 (95% CI: 0.10–0.73) episodes per 100 child-months (5/185 AGE episodes) and the prevalence of norovirus was 4.6% (13/282 stool samples collected). In the hospital-based surveillance cohort, at least one pathogen was detected in 50% of samples (44/88 stool samples collected) and norovirus prevalence was 6.8% (6/88 stool samples collected).

**Discussion:**

This report demonstrates how the occurrence of the COVID-19 pandemic hindered the conduct of clinical trials. However, this also created unique research opportunities to investigate the potential impact of pandemic control measures on the etiology of infectious diarrheal disease.

## 1 Introduction

Norovirus is a highly transmissible pathogen, and norovirus infection is among the most common cause of acute gastroenteritis (AGE). It accounts for approximately 18% of all diarrheal disease worldwide and is a major contributor to morbidity and mortality (1–3). Norovirus infection is responsible for approximately 200,000 deaths globally per year, with ≥70,000 of these being reported in children in developing countries (1, 4). Therefore, investigation into strategies, including vaccination, to effectively control norovirus in the wider community is imperative. However, given the challenges in developing an effective norovirus vaccine, setting up surveillance activities prior to initiating clinical trials that evaluate disease prevention strategies can help to prepare trial sites and generate valuable input on study design and sample size estimations (5, 6). In January 2020, we began enrolling children aged <2 years into an epidemiological study in Panama to estimate the burden of norovirus in preparation for evaluating upcoming prevention strategies, including vaccination.

Unfortunately, our study was disrupted by the onset of the coronavirus disease 2019 (COVID-19) pandemic in March 2020, with the first COVID-19 case reported in Panama on March 9 2020 (7), and the subsequent implementation of COVID-19 disease transmission control procedures (8). Several studies have documented the impact of COVID-19 mandates on the incidence and reporting of other common infectious diseases, including influenza, respiratory syncytial virus, and streptococcal pharyngitis (9–11). Here, we discuss the challenges faced in conducting an epidemiological study evaluating the burden of norovirus in children aged <2 years in Panama during the COVID-19 pandemic. We also report ancillary findings related to the incidence, prevalence, and etiology of AGE caused by norovirus upon revising our study methodology to comply with mandates to limit COVID-19 exposure.

## 2 Materials and methods

The study was composed of two parts: an observational, longitudinal, community-based AGE surveillance study conducted in two provinces in Panama (Panama and Chiriquí) and a hospital-based AGE surveillance study conducted concurrently in Panama City.

The study methodology for both cohorts is summarized in Supplementary Table S1. For the longitudinal study, healthy children aged 5‒18 months were enrolled from January 6 through March 23, 2020 at the study sites and actively followed for a period of approximately 6 months. The last participant was contacted on September 23, 2020. Sample size calculations indicated that a total of 480 children (240 at each province) would be optimal to estimate the norovirus-related AGE annual incidence rate. Surveillance included weekly contact through a mobile application or telephone to identify potential AGE episodes. Participant's parents were asked to confirm occurrence of any symptoms and to collect stool samples in the first 7 days after symptom onset. Those who reported an AGE episode were prompted to visit the surveillance clinic, or alternatively, surveillance staff performed a home visit. Incidence of AGE (all cause and norovirus attributable) and severity of symptoms were determined.

For the hospital-based study, starting on January 21, 2020, children aged <2 years who were admitted to the pediatric service or emergency room at the Hospital del Niño Dr. José Renán Esquivel in Panama City due to AGE were evaluated. The last sample was collected on September 29, 2020 and the database was locked on November 30, 2021. Sample size calculations indicated that at least 180 children hospitalized or attending the emergency room would be required to estimate the norovirus-attributable fraction of AGE. Stool samples were collected within 48 h of hospital admittance.

For both cohorts, AGE was considered norovirus-related if the participant presented: ≥3 loose or liquid stools; and/or ≥2 episodes of vomiting; or ≥1 episode of vomiting plus ≥1 liquid stool in any 24-hour period; as well as a norovirus-positive stool sample by real-time reverse transcriptase polymerase chain reaction (RT-PCR) [GI/GII Norovirus Multiplex (TaqMan®) RT-PCR Assay] (12) tested at a designated laboratory. In addition, stool samples were tested for astrovirus and sapovirus using multiplex real-time RT-PCR as has been previously reported (13), and for enteropathogens including *Campylobacter*, *Clostridium difficile*, *Escherichia coli*, *Salmonella*, *Shigella* (*S. boydii*, *S. sonnei*, *S. flexneri*, and *S. dysenteriae*), *Vibrio cholerae*, adenovirus, rotavirus, *Cryptosporidium* (*C. parvum* and *C. hominis* only), *Entamoeba histolytica*, and *Giardia lamblia*, including co-infections with norovirus (xTAG® Gastrointestinal Pathogen Panel, Luminex, USA). Participants enrolled in the longitudinal community-based study were not duplicated in the hospital study.

The study was conducted according to Good Clinical Practice, the principles of the Declaration of Helsinki, and the codes and regulations of the participating countries regarding research on human subjects. The study was approved by the necessary ethical review boards and as locally required by the regulatory authorities of Panama (Comité de Bioética en Investigación del Hospital del Niño Dr. José Renán Esquivel). Parents provided written informed consent for their children.

Due to the COVID-19 pandemic, a series of Executive Decrees from the Ministry of Health for curfew and migratory restrictions were implemented in Panama. From March 24, 2020 the curfew was extended to 24 h a day and from March 30, 2020 migratory restrictions based on sex and national ID card number were implemented in metropolitan areas; these restrictions were extended throughout Panama on June 6, 2020. Migratory restrictions were lifted on September 11, 2020 but were reinstated from December 28, 2020 through January 14, 2021 (8). As a result, adjustment of clinical study protocols was needed to account for pauses in recruitment, in-person data collection, safety assessments, and investigational drug treatments (14, 15).

As a result, all domiciliary and site visits for our study were interrupted. A contingency plan was implemented for stool sample collection; health personnel were authorized to collect stool samples from a participant's home and/or participants could take samples to the study sites at the designated time permitted by the health authorities. In addition, the number of hospitalizations in 2020 decreased by 250 per month in children <2 years of age, which impacted recruitment into the hospital-based study, necessitating sample size adjustments and a reduced power to estimate the attributable fraction of AGE due to norovirus. Consequently, our efforts to collect a complete epidemiological dataset and infer the burden of norovirus among children in Panama were hampered by the COVID-19 pandemic. We did, however, note interesting ancillary findings worth reporting and these are described herewith.

## 3 Results

Children in the longitudinal surveillance cohort [*N *= 400 (*n* = 239 from Chiriquí and *n* = 161 from Panama); recruited from January 6, 2020, through March 23, 2020] were followed for a median of 5.9 months resulting in a total study period of 1594.6 child-months. A total of 185 AGE episodes were documented (*n* = 100 from Panama and *n* = 85 from Chiriquí), resulting in an overall AGE incidence of 11.6 (95% CI: 9.99–13.4) episodes per 100 child-months [11.4 (95% CI: 9.2–13.8) in Panama and 11.9 (95% CI: 9.5–14.7) in Chiriquí]. Of these, 54.6% (*n *= 101) of cases were reported through the mobile app surveillance system (with information being verified and reconciled with physician follow-up) while the remainder were captured via telephone. The number of AGE episodes with results was 128 (*n* = 73 from Panama and *n* = 55 from Chiriquí).

**Figure 1:**
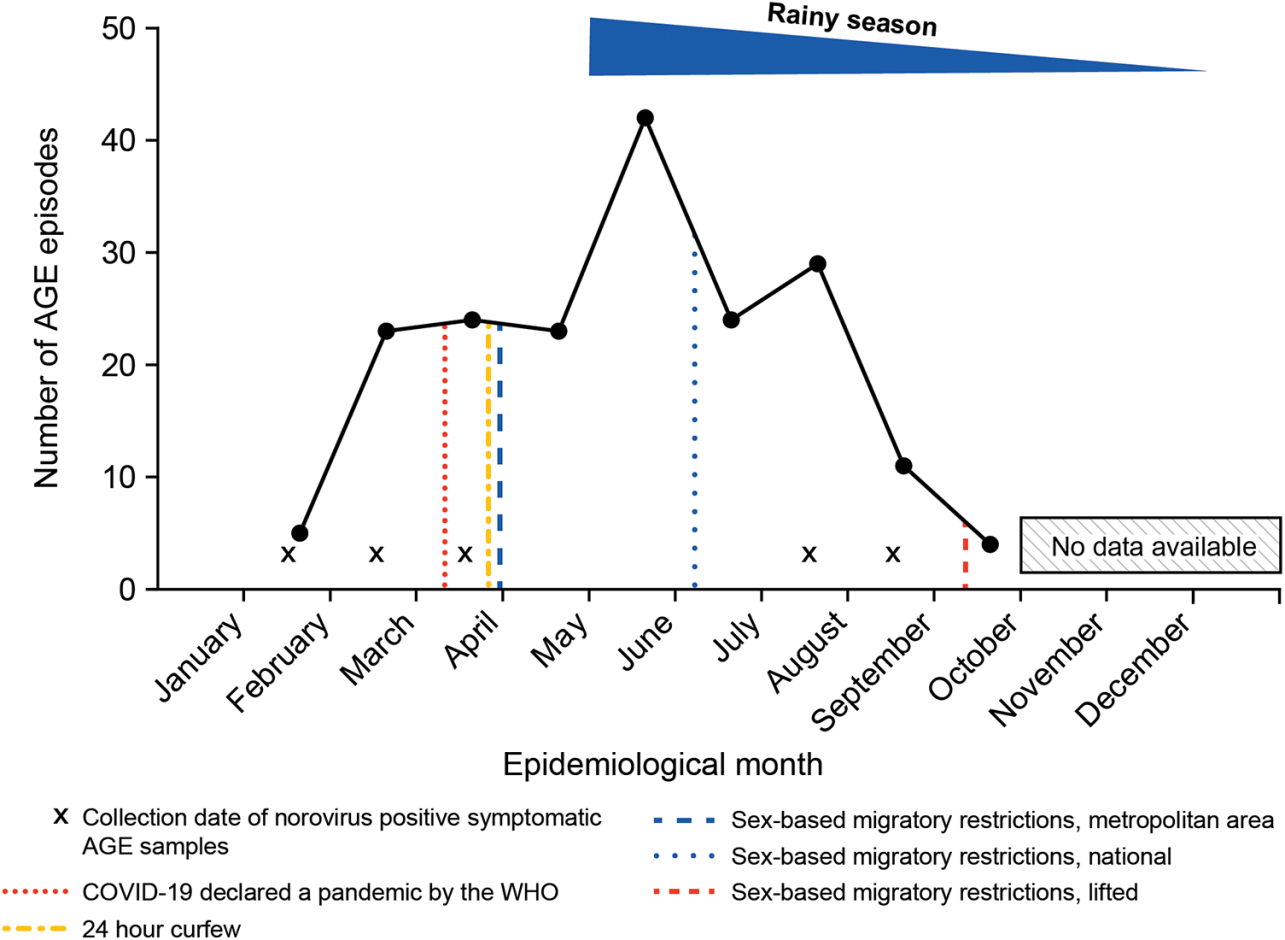
Number of AGE episodes* from children enrolled in the longitudinal surveillance cohort (*N *= 400) in relation to physical distancing mandates in Panama during 2020. AGE, acute gastroenteritis. *Total number of AGE episodes = 185.

The peak occurrence of AGE episodes was in May at the beginning of the rainy season, after implementation of sex-based migratory restrictions in the metropolitan area of Panama but before national implementation (Figure 1). The study was completed before the last trimester of 2020, which marks the end of the rainy season and follows lifting of the sex-based migratory restrictions. In this cohort, 5/185 AGE episodes had norovirus as an etiologic agent, resulting in a norovirus-related AGE incidence of 0.3 (95% CI: 0.10–0.73) episodes per 100 child-months [0.6 (95% CI: 0.18–1.32) in Panama]. Norovirus genotype was determined, with four samples genotyped as GII and one as GI. Two of these stool samples also tested positive for other pathogens (*C. difficile* and *Campylobacter, and Sapovirus*).

In the hospital-based surveillance cohort, 88 children were evaluated and provided stool samples. At least one pathogen was detected in 50% of samples (*n* = 44) and norovirus prevalence was 6.8% (*n* = 6) (Table 1). All norovirus-positive samples were the GII genotype. Of the norovirus-positive stool samples, two were also positive for other pathogens (*Campylobacter* or adenovirus). At least one pathogen was detected in 136/370 (36.8%) stool samples collected from AGE symptomatic children across the longitudinal and hospital surveillance cohorts (Table 1). *C. difficile*, *Campylobacter*, and norovirus occurred most frequently in the pooled cohorts.

**Table 1:** Enteropathogens (AGE etiology) detected in stool samples from symptomatic patients across longitudinal and hospital surveillance cohorts in Panama in 2020.

Enteropathogens detected, *n* (%)	Surveillance cohort		
	Longitudinal (*N* = 282)	Hospital (*N* = 88)	Total (*N* = 370)
≥1 detected	92 (32.6)	44 (50.0)	136 (36.8)
Virus			
Norovirus	13 (4.6)	6 (6.8)	19 (5.1)
Sapovirus	4 (1.4)	0	4 (1.1)
Adenovirus	1 (0.4)	7 (8.0)	8 (2.2)
Astrovirus	1 (0.4)	0	1 (0.3)
Bacteria			
*Campylobacter*	24 (8.5)	20 (22.7)	44 (11.9)
*C. difficile*	26 (9.2)	2 (2.3)	28 (7.6)
*Salmonella*	8 (2.8)	8 (9.1)	16 (4.3)
*Shigella*	9 (3.2)	3 (3.4)	12 (3.2)
Enterotoxigenic *Escherichia coli*	7 (2.5)	1 (1.1)	8 (2.2)
*Escherichia coli*	2 (0.7)	0	2 (0.5)
Parasite			
*Giardia lamblia*	7 (2.5)	3 (3.4)	10 (2.7)
Norovirus + ≥1 other pathogen detected	2 (0.7)^a^	2 (2.3)^b^	4 (1.1)

*N*, total number of stool samples in each cohort; *n*, number of stool samples within each descriptor. AGE, acute gastroenteritis.

^a^ *Sapovirus*, *Camplobacter and C. difficile*.

^b^ *Campylobacter* and Adenovirus 40/41.

## 4 Discussion

The COVID-19 pandemic and policies implemented to reduce its spread caused considerable disruption to day-to-day healthcare, including collection of clinical data and access to treatment. Key findings from a systematic review show that delayed enrollment and operational gaps in most ongoing clinical trials had a negative impact on trial programs and data integrity (16, 17). Having made amendments to our epidemiological study, we found that patient recruitment, AGE, and ability to fulfill treatment recommendations were affected. In addition, although a mobile app and telephone contact were available to collect information on AGE symptoms, only 16%–42% of symptom severity information was available for fever, diarrhea, vomiting, behavior, and dehydration, which could potentially be attributable to the lack of in-person follow-up. Delays in stool sample collection following AGE onset may have also impacted viral detection. Furthermore, although the physical distancing policies included exceptions related to “health reasons”, it was documented qualitatively that some participants were inhibited or delayed in accessing treatment for dehydration. This precluded us from accurately inferring the burden of norovirus in children living in Panama.

Nevertheless, discussion of our data on norovirus outcomes in the context of AGE symptoms during the COVID-19 pandemic is warranted. In our longitudinal surveillance study, we found an overall norovirus AGE incidence rate of 0.3 episodes per 100 child-months. Across Latin America and in Panama specifically, the frequency of norovirus detected in stool samples collected from children with AGE has been shown to be approximately 15% and 37%, respectively (18, 19). The highest number of AGE episodes occurred at the beginning of the rainy season. As reported by others, cases of norovirus are more often associated with the rainy season in tropical countries (20, 21). However, it is not unusual for cases to also occur during the dry season as a result of specific outbreaks caused by tourism or school-related activities (22). Across the longitudinal and hospital-based surveillance cohorts, we noted that viral causes of AGE were relatively low (32/370; 8.6%) while bacterial causes of AGE were higher (110/370; 29.7%). Previous studies identified norovirus and rotavirus as the most frequent viral causes of AGE in children aged 1 month to 15 years of age (18). The recent introduction of the rotavirus vaccination has reduced rotavirus-associated AGE; however, diarrhea caused by norovirus has risen becoming the leading cause of hospitalization in the pediatric population, especially in countries that introduced rotavirus vaccines (23). Rotavirus vaccine coverage is estimated to be ∼86% in the Panama region (24, 25) and, in this study, rotavirus was not detected in stool samples of any participants with AGE.

Others studying norovirus during the COVID-19 pandemic found that norovirus prevalence was reduced. For example, in the UK, a substantial and sustained reduction in norovirus outbreaks and norovirus-positive laboratory reports to Public Health England was noted; this was suggested to be a consequence of decreased norovirus reporting (84.6%) as well as a decline in the referral of norovirus-positive samples for genotyping (26). Similar findings have been observed in other countries including Australia, Germany, China, and the USA following implementation of COVID-19 control measures (27–30). The COVID-19 pandemic also resulted in a marked reduction in the number of reported gastrointestinal infections (31). These results are likely multifactorial, with reduced transmission (typically through person-to-person contact or contaminated food and water) being partially accountable due to mandated mobility restrictions and individual behavioral changes, such as an increase in hygiene and sanitary care at the family and community level. Indeed, reduced incidence and prevalence of other viral infections transmitted from person-to-person contact, such as respiratory syncytial virus and influenza, have also been reported (11, 32, 33). In our setting, social distancing measures led to a greater reduction in viral than in bacterial gastroenteritis. A study in Southern China found that the incidence of norovirus-related AGE was significantly higher in 2020, following relaxing of interventions to stop COVID-19 transmission (34). This highlights the importance of continued surveillance for viral gastroenteritis post pandemic.

This study did not sample SARS-CoV-2 in participants' stool samples, so no inference of the biological interaction with the other viruses present can be made. This report demonstrates how the occurrence of the COVID-19 pandemic hindered the conduct of clinical trials, including this epidemiological study to estimate the burden of norovirus in Panama, causing logistical challenges in participant enrollment and follow-up. This will likely have a further impact on the development of norovirus prevention strategies, including vaccines.

## Manuscript contribution summary

Norovirus is a highly contagious pathogen. Infection with this virus is one of the leading causes of diarrheal disease worldwide and is a major-contributor to disease-related deaths. Development of vaccines, to help prevent disease, are an important part of an effective norovirus control strategy. However, understanding the distribution of norovirus in the population is important to assist with vaccine clinical trial design and implementation. This surveillance study was initiated in January 2020 and investigated cases of norovirus-related acute gastroenteritis in both a community- and hospital-based setting. Unfortunately, this study was disrupted by the coronavirus disease 2019 (COVID-19) pandemic and the methodology was revised to comply with the implementation of disease transmission control procedures. In this manuscript, we discuss the challenges faced in conducting an epidemiological study evaluating the burden of norovirus in children aged <2 years in Panama during the COVID-19 pandemic. We also report additional findings showing the incidence, and prevalence of acute gastroenteritis caused by norovirus. This report demonstrates how the occurrence of the COVID-19 pandemic hindered the conduct of clinical trials. However, this also created unique research opportunities to investigate the potential impact of pandemic control measures on the causes of infectious diarrheal disease.

## Data Availability

The datasets presented in this article are not readily available because the datasets, including the redacted study protocol, redacted statistical analysis plan, and individual participants’ data supporting the results of the completed study, will be made available within three months from initial request, to researchers who provide a methodologically sound proposal. The data will be provided after its de-identification, in compliance with applicable privacy laws, data protection and requirements for consent and anonymization. Requests to access the datasets should be directed to John Weil, john.weil@takeda.com and Rodrigo DeAntonio, rodrigo.deantonio@cevaxin.com.
