# Supplementary material for: Norovirus in children under 2 years of age: an epidemiological study in Panama during the COVID-19 pandemic
Source: Front Pediatr. 2024 Feb 15;12:1292967. doi: 10.3389/fped.2024.1292967 (PMC10902054; doi:10.3389/fped.2024.1292967)
Supplement: Supplementary file 1 [file Table1.docx]

**Supplementary Table 1. Summary of study methodology for the longitudinal and hospital surveillance cohorts**

|  | **Surveillance cohort** | |
| --- | --- | --- |
|  | **Longitudinal** | **Hospital** |
| **Enrolled population** | Children 5-18 months of age and resident of Panama or Chiriqui provinces | Children <2 years of age admitted to the Hospital del Niño Dr. José Renán Esquivel in Panama City due to AGE |
| **Obtained written informed consent** | Yes | Yes |
| **Enrollment period** | January 6, 2020 – March 23, 2020 | January 21, 2020 – September 29, 2020 |
| **Estimated sample size** | 480 participants | 180 participants |
| **AGE surveillance data collection** | Weekly contact via mobile application or telephone | Data collected from patients admitted to the pediatric service or emergency room due to AGE |
| **Stool samples** | Collected ≤7 days from symptom onset | Collected ≤48 hours of hospitalization |
| **Date of data collected from last participant** | September 23, 2020 | September 29, 2020 |
